# Treatment efficacy for pain complaints in women with endometriosis of the lesser pelvis after laparoscopic electroablation vs. CO_2_ laser ablation

**DOI:** 10.1007/s10103-014-1630-4

**Published:** 2014-07-23

**Authors:** Ewa Posadzka, Robert Jach, Kazimierz Pityński, Marcin Jacek Jablonski

**Affiliations:** 1 Departament of Gynecology and Obstetrics, Jagiellonian University Medical College, 23 Kopernika str, 31-501 Krakow, Poland; 2 Jesuit University Ignatianum in Krakow, 26 Kopernika str, 31-501 Krakow, Poland

**Keywords:** Laser CO_2_ vaporization, Electroablation, Endometriosis, Dyspareunia

## Abstract

Endometriosis is a chronic disease affecting mainly women of the reproductive age. Its most common manifestations include impaired fecundity, pelvic pain, and dyschezia. Laparoscopic removal of endometriotic foci remains to be the gold standard for the treatment of endometriosis. More effective techniques of endoscopic approach—among others, laser application—are continually being developed. The aim of the study was to evaluate the efficacy of laparoscopic treatment with the use of CO_2_ laser ablation vs. electroablation with regard to pain complaints in the affected patients. The study included 48 women (aged 22–42) with varying degrees of endometriosis of the lesser pelvis. The Numeric Rating Scale (NRS) was used to evaluate pain intensity before the surgery in all patients, followed by either laser ablation or electroablation of the endometriotic foci. The results of the laparoscopic treatment were monitored after 3 and 6 months postoperatively. *p* value of 0.05 was considered to be statistically significant. Patients from both groups reported less intensive pain before/during menstruation (dysmenorrhea) 6 months postoperatively, with more distinct tendency in the electroablation group (*p* = 0.004) as compared to the laser ablation group (*p* = 0.025). Despite the initial improvement reported at the 3-month checkup (*p* = 0.008), 6 months postoperatively, a statistically significant increase in pain intensity was noted in both groups (*p* = 0.016 and *p* = 0.032 for CO_2_ laser ablation and electroablation, respectively). Both surgical methods seem to be effective only in the treatment of endometriosis-related dysmenorrhea, whereas the intensity of other pain complaints (dyspareunia, dysuria, dyschezia, pelvic pain syndrome (PPS)) has remained on the same level.

## Introduction

Endometriosis is an estrogen-dependent chronic disease with the incidence of 30–40 % in women with pain complaints of the lesser pelvis [1, 2]. The disease is estimated to affect 10–15 % of asymptomatic women of the reproductive age [3]. Endometriosis is connected with the presence of ectopic endometrial cells within the organs of the lesser pelvis and with biochemical, molecular, and hormonal changes which negatively affect the homeostasis in a female body. Endometriotic women are known to have elevated levels of macrophages [4], dendritic cells [5], and peritoneal fluid NK cells as well as abnormal levels of inflammatory mediators, i.e., interleukin-1 (IL-1), Il-6, IL-8, substance P, and tumor necrosis factor (TNF). Disturbances in the functioning of the immune system lead to chronic inflammatory processes [6] and are associated with the appearance of the following symptoms of inflammation: dysmenorrhea, dysuria, and dyspareunia. The presence of the abovementioned manifestations is connected with the pathogenesis of endometriosis as well as the consequences of an inflammation, namely intraperitoneal adhesions [7]. The foci of ectopic endometrial cells may develop within the ovaries, fallopian tubes, corpus uteri, Douglas pouch, vesicouterine pouch, as well as outside the pelvis, for example on the surface of colon, parietal peritoneum, and abdominal tissue. The most common, nonspecific endometriosis-related complaints include dysmenorrhea, dyspareunia, dysuria, dyschezia, and noncyclic (not related to menstrual cycle) chronic pelvic pain. The pain may be correlated with the advancement of the endometrial lesions, but only in grades III and IV cases, whereas grades I and II have not been confirmed to be related [8, 9].

Endometriosis is clinically graded into four stages of advancement according to the revised American Society of Reproductive Medicine classification. The Chapron classification (2003) is an example of another system, which introduced the deep infiltrating endometriosis (DIE) term as the main cause of pain complaints, depending on the location within the organs of the lesser pelvis [10]. Apart from DIE, endometriosis can be staged into ovarian and peritoneal.

The consequences of the disease are a serious and complex social issue, although it is often underestimated or ignored. Endometriosis is a multifactorial disease [11] and extends beyond a mere medical condition, resulting in impaired fecundity, disturbed family relations, and leaves of absence from work due to chronic pain complaints.

Laparoscopy remains to be the gold standard in the diagnosis and treatment of endometriosis [12]. Enucleation or destruction of cysts, removal or destruction of the focal lesions, lysis of adhesions, and/or the nerve-sparing procedure of selective laparoscopic uterine nerve ablations (LUNAs) of the uterosacral ligaments belong the most common laparoscopic methods applied in the treatment of the disease. These techniques aim to maximize the therapeutic effect and minimize the side effects, i.e., damage to the healthy tissue. CO_2_ laser ablation seems to be the most beneficial of them all, as it allows for a precise vaporization of endometriotic lesions with good homeostasis and minimal damage to surrounding tissue. The latter is especially important in the event of a removal of the endometrial cysts within the ovary, as it allows preservation of the ovarian reservoir [13, 14].

The efficacy of laser ablation of endometrial implants to reduce pain is estimated at almost 65 % [15]. However, there is no consensus on the positive therapeutic effect of other classical laparoscopic techniques such as electroablation [16]. Latest research shows that laparoscopy with surgical management of the focal lesions has a beneficial therapeutic effect and lowers the possibility of recurrence of endometrial cysts and complaints. It also increases the chances of conception [17].

### Objectives

The aim of the study was to evaluate the therapeutic effect of two laparoscopic techniques, electroablation of endometriosis vs. CO_2_ laser ablation, to reduce a variety of pain complaints in the affected individuals.

## Material and methods

A laser is a device that emits electromagnetic radiation in various ranges of wavelengths: visible light, ultraviolet, or infrared radiation, by using stimulated emission. The name is an acronym of the *light amplification by stimulated emission of radiation*.

Laser radiation is coherent, usually polarized, and the beams have very small divergence. Narrow emission linewidth, which corresponds to a high power in a selected, narrow spectrum range, may be easily generated. The CO_2_ laser is a gas laser based on a gas mixture of carbon dioxide, nitrogen, hydrogen, and helium. The main spectral lines are within the wavelength of 9.4 and 10.6 μm, and the emitted power reaches 100 kW at continuous and 10^13^ pulsed operation. The AcuPulse CO_2_ laser of 30–40 W (Lumenis, Israel), connected to a laparoscope (Storz, Germany), and Aida software (Storz, Germany) to archive data were used in the study.

A total of 48 patients with ovarian endometriosis and DIE, grades I (*n* = 0), II (*n* = 4), III (*n* = 37), and IV (*n* = 7) according to rARSM, prepared for laparoscopic removal of endometriotic lesions at the Clinic of Gynecology and Oncology, Kraków, between 2011 and 2012, were recruited for the study. Exclusion criteria were the following: pregnancy, history of surgery within the abdominal cavity, history of pelvic inflammatory disease, and prolonged use of oral contraceptives. Before the surgery, all patients filled a questionnaire on pain complaints during the 3 months preceding the study. Numerical Rating Scale (NRS) was used in the questionnaire, with 0 for “no pain” and 10 for “unbearable pain,” to evaluate the intensity of five types of pain during menstruation, urination, defecation, sexual intercourse, as well as noncyclic pelvic pain syndrome (PPS). The respondents were also asked to submit information on their use of painkillers. Patients deemed eligible for the study underwent a gynecological examination and an ultrasound test with the use of transvaginal probe, 2D/3D/4D 5–9 MHz, Voluson E6 (General Electrics, USA). All women underwent laparoscopic destruction of the endometriotic foci. The subjects were randomized into the laser ablation (*n* = 15) and electroablation (*n* = 33) groups. At the 3- and 6-month checkup, the patients completed the pain questionnaire again. Additionally, changes in painkiller use were also investigated. At the first visit, when patients deemed eligible for the study were selected and all control checkups, the subjects were examined with the use of a transvaginal ultrasound to evaluate the presence of endometrioid lesions. The tests were performed by the same experienced and certified (ISUOG, PTG, IOTA) doctor, Agnieszka Nocuń.

### Statistical analyses

Student’s *t* test for matched pairs (for near-normally distributed variables) and nonparametric Wilcoxon test (for no-normally distributed variables) were used to evaluate changes in pain intensity. McNemar’s test was used to analyze the percentage of patients using antispasmodic drugs (e.g., No-Spa—drotaverine hydrochloride) and nonsteroidal anti-inflammatory drugs (NSAIDs) (the most commonly used). Student’s *t* test for independent groups and Mann-Whitney *U* test for nonnormally distributed variables were used for the comparison between changes in pain intensity inpatients with disease recurrence vs. no recurrence. *p* < 0.05 was accepted as statistically significant. Microsoft Office Excel and SPSS Statistics 17.0 were used for the calculations. The study was approved by the Ethics Committee of Collegium Medicum, Jagiellonian University.

## Results

A total of 48 patients were deemed eligible for the study, with 33 women who underwent electroablation (group characteristics, Table 1) and 15 subjects who received CO_2_ laser ablation (group characteristics, Table 2). Out of the 48 patients who underwent surgery, five (10.42 %) conceived postoperatively and were excluded from further analysis. Three (6.25 %) women withdrew from the study without stating the cause. At the 3-month checkup, the electroablation and laser ablation groups comprised 25 (75 %) and 15 (100 %) subjects, respectively. Eventually, the 6-month checkup included 34 (71 %) patients, 20 (60 %) after electroablation and 14 (93 %) after CO_2_ laser ablation. One (2.9 %) woman conceived and five (14.7 %) resigned from the 6-month checkup.

**Table 1:** Patient characteristics: electroablation group

No. of patients (*n* = 33)	Mean	Median	Min. value	Max. value
Age	30.91	30	23	42
BMI	21.67	21.61	17.65	27.92
Dysmenorrhea	6.03	7.00	0	10
Dyschezia	1.09	0.0	0	8
Dysuria	1.06	0.0	0	8
PPS	2.46	2.97	0	10
Dyspareunia	2.94	3.0	0	9

**Table 2:** Patient characteristics: CO_2_ laser ablation group

No. of patients (*n* = 15)	Mean	Median	Min. value	Max. value
Age	30.17	31	22	39
BMI	20.97	17.75	20.89	24.65
Dysmenorrhea	6.27	7.00	0	10
Dyschezia	0.93	0.0	0	7
Dysuria	1.87	0.0	0	10
PPS	3.6	2.82	0	8
Dyspareunia	3.87	4.0	0	7

The statistical analyses generated diversified results. In the CO_2_ laser group, the 3-month checkup revealed statistically significant pain relief only during sexual intercourse (*p* = 0.035). Pain threshold increased in one (6.67 %), decreased in nine (60 %), and remained on the same level in five (33.3 %) patients. In the electroablation group, the 3-month checkup revealed statistically significant pain relief during menstruation (*p* = 0.01), urination (*p* = 0.037), and sexual intercourse (*p* = 0.008). Dysuria decreased from seven to four points in 7 (28 %), remained on the same level in 16 (64 %), and increased in 2 (8 %) patients, whereas 12 (50 %) subjects reported complete resolution of pain symptoms.

**Fig. 1:**
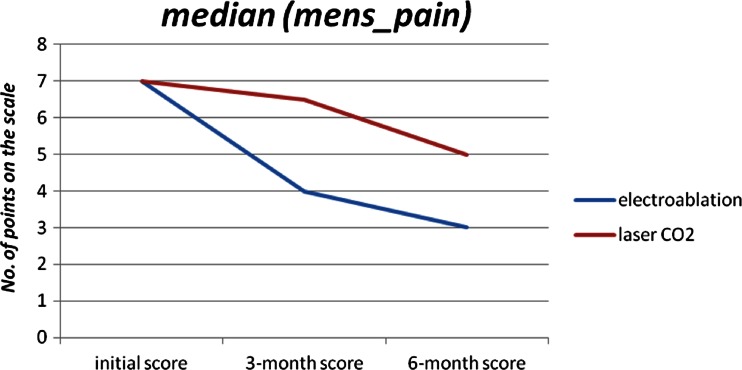
Changes in pain complaints before/during menstruation (electroablation vs. CO_2_ laser ablation), expressed in points, before the operation, 3 and 6 months after the surgery

**Fig. 2:**
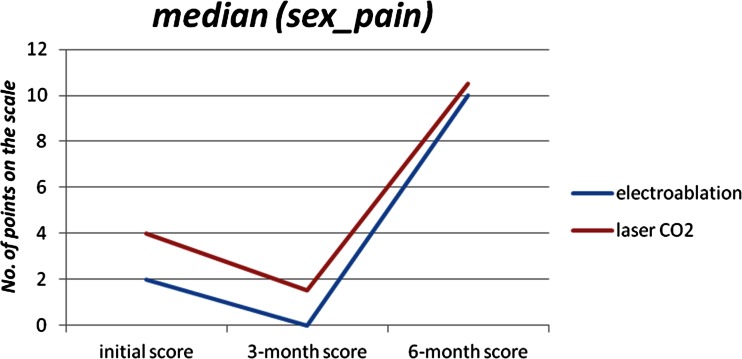
Changes in pain complaints during sexual intercourse (electroablation vs. laser ablation), expressed in points, before the operation, 3 and 6 months after the surgery

The 6-month checkup revealed further changes. In the CO_2_ laser group, dysmenorrhea significantly subsided (*p* = 0.025), but dyschezia intensified (*p* = 0.018). Fifteen (100 %) patients presented no dyschezia complaints before the surgery, but after 6 months, seven (50 %) patients reported pain of mild intensity. Interestingly, after the initial improvement observed at the 3-month checkup, the 6-month checkup revealed significantly intensified dyspareunia (*p* = 0.016). Mean values increased from 3.75 to 9.38 (Fig. 2). In the electroablation group, pain related to menstruation continued to decrease (*p* = 0.004) (Fig. 1). Seventeen (50 %) patients reported dysmenorrhea at the level of greater than or equal to seven points before the surgery, but four and three points at the 3- and 6-month checkup, respectively. However, dyspareunia intensified significantly (*p* = 0.032) in that group. Seventeen (50 %) patients reported dyspareunia at the level of greater than or equal to two points before the surgery, but greater than or equal to ten points at the 6-month checkup.

No statistically significant changes in terms of PPS (*p* > 0.05) were observed in either of the investigated groups. The frequency of the use of painkillers and antispasmodic drugs was also analyzed in both groups. NSAIDs (ketonal, paracetamol) and antispasmodic drugs (No-Spa) were most often used. Statistical analysis was carried out for both of these groups of drugs, and statistically significant decrease in the use of NSAIDs was noted in both study groups (*p* = 0.008 for electroablation and *p* = 0.031 for laser ablation), but not sooner than 6 months postoperatively. The frequency of postoperative use of antispasmodic drugs was comparable to preoperative values (*p* > 0.05).

Owing to the diversified results from both study groups, a relation between the investigated types of pain and disease recurrence on transvaginal ultrasound (*n* = 7) was investigated. Recurrence was noted only in the laser ablation group. A statistically significant correlation (*p* = 0.02) was found between intensified dyschezia and disease recurrence at the 6-month checkup (Table 3) in that group of patients.

**Table 3:** Analysis of correlations between pain intensity during sexual intercourse and disease recurrence on ultrasound

Type of pain	Usg_recidiv_6 m	No. of patients	Mean	Median	Standard deviation	Min. value	Max. value	*p* value
Defec_pain_6-m laser	0	7	1.00	0.00	1.732	0	4	0.020
	1	7	3.86	5.00	2.410	0	6	

No correlations between changes in pain intensity and disease recurrence on ultrasound within the lesser pelvis (*p* > 0.05) with regard to the remaining types of pain were found.

## Discussion

Pathogenesis of pain in endometriosis remains to be a complex issue. Regardless of various theories, for example histologic comorbidity of endometriotic foci and the ends of nerve fibers [19], changes in TNF gene expression [18], smooth muscle hyperreflexia and hyperalgesia of interstitium of endometriotic lesions [20], and excessive innervation of endometrioid ovarian cysts [21], there is no consensus regarding the pathogenesis of pain complaints and their diversification. Abdominal (nociceptive) pain, caused among others by chronic inflammation and endometrial infiltration into the healthy tissue, is the main component of endometriosis-related pain, while nonnociceptive pain (hyperalgesia, psychogenic factors) is of less significance. Due to the multifactorial nature of pain pathogenesis in endometriosis, the “gold standard” is yet to be established. Thus, the search for optimal patient management, mostly based on prospective studies, continues. Apart from surgical removal of the endometriotic lesions, pharmacotherapy (hormonal contraceptives, gonadotropin agonists) remains to be the most commonly used alternative method of pain management, whereas vagus nerve stimulation [22], diet therapy, or alternative medicine [23] are less popular.

Our investigation generated diversified findings. It seems that both CO_2_ laser ablation and electroablation result in relief of pain complaints before and during menstruation. Although it is not a disease-specific symptom, dysmenorrhea is a common complaint among the affected women. Fauconnier et al. [24] mentioned it in their study, while Hsu et al. [9] suggested a lack of correlation between pain complaints and location of endometriosis. Significant decrease in mean pain intensity and, more importantly, a tendency to further drop after 6 months since the surgery allow to draw optimistic conclusions about the therapeutic effect of both procedures [25] (Fig. 1). Our results are consistent with the findings of a randomized prospective study by Sutton et al. [26] and Jacobson [27] review, with regard to laser ablation.

Similar results have been reported by Radosa et al., who investigated the effectiveness of electroablation [28], in their retrospective study over the course of 2 years, and also by Roman [29] in prospective study of the effects of electrosurgical excision.

Also, it is noteworthy that the use of NSAIDs, associated mostly with dysmenorrhea-related pain, was also statistically significantly decreased (*p* < 0.08 after 6 months).

Regardless, changes in the intensity of the remaining types of pain do not allow for unambiguous assessment of the positive effects of laparoscopic treatment. Both CO_2_ laser ablation and electroablation resulted in intensified dyspareunia 6 months postoperatively (Fig. 2) and, especially in the former group, significantly increased dyschezia. Intensified pain complaints in these cases might have been the result of the surgical technique of removing endometriotic lesions from the areas of the Douglas pouch and uterosacral ligaments, as both of these locations are associated with these two types of pain [30, 31]. Previous surgical techniques of removing DIE from the area of uterosacral ligaments deemed to be positive therapeutic results in terms of subsiding dyspareunia and dysmenorrhea, but they also increased the number of grave urologic complications due to nerve damage around the bladder and consequently resulted in the necessity of self-catheterization in some patients [32, 33]. The precision of CO_2_ laser, believed to be the essential factor for preservation of the ovarian reserve in the treatment of ovarian endometriosis, may have a less beneficial therapeutic effect in DIE therapy of the Douglas pouch and promotes earlier recurrence of endometriosis and, consequently, pain complaints, especially dyspareunia [34, 35]. Meuleman et al. [36] obtained good results of DIE treatment with the use of CO_2_ laser, but the study included patients with DIE of the rectosigmoid area and management consisting of segmental colon resection with the additional use of CO_2_ laser.

Evaluation of partial therapeutic effect ought to take into account hyperalgesia of the nerve endings of tissues theoretically neighboring DIE lesions [37], which in case of electroablation and laser ablation, probably are less damaged than by the radical surgical approach. As a result, disease recurrence takes place faster than after the classical approach, i.e., excision of the endometriotic lesions with a margin of healthy tissue. Such an explanation is consistent with the results of the abovementioned study by Meuleman et al. Paradoxically, the precision of CO_2_ laser, believed to be its advantage in the treatment of ovarian endometriosis, might not correspond to the long-term therapeutic effect in pain management of extra ovarian endometriosis, i.e., DIE.

## Conclusions

Pain relief in patients after laparoscopic CO_2_ laser ablation and electroablation is short-term and incomplete. Precision of the CO_2_ laser and relatively limited tissue damage during electroablation seem to be irrelevant when it comes to their practical application in treating pain related to extra ovarian endometriosis. Owing to a short postoperative observation period, our results should be regarded as a preliminary evaluation of treatment efficacy for pain complaints [38]. Further prospective analysis is necessary, and additionally, long-term hormonal therapy as adjuvant to laparoscopic treatment ought to be considered [39].
